# Research or teaching? That is the problem: A narrative inquiry into a Chinese college English teacher’s cognitive development in the teaching-research nexus

**DOI:** 10.3389/fpsyg.2023.1018122

**Published:** 2023-02-02

**Authors:** Hui Ni, Xinxin Wu

**Affiliations:** ^1^School of Foreign Languages, Heze University, Heze, China; ^2^School of Foreign Studies, Jiangnan University, Wuxi, China

**Keywords:** teaching-research nexus, cognition, mediation, researcher, professional development

## Abstract

This narrative inquiry traces a Chinese college English teacher’s cognitive transformation in the teaching-research nexus over a 4-year time period from the perspective of sociocultural theory (SCT). Several stories were narrated based on the data from reflective journals, literature reading notes, and interaction notes to show how the participant walked out of the teaching-research contradiction and finally achieved professional development. The findings indicate that the college English teacher’s perception of the research-teaching nexus developed when she actively exercised her agency to take part in social interactions in a supportive sociocultural environment with the regulation of appropriate mediational tools. The conceptual progress in the research-teaching nexus finally contributed to her professional development in both teaching and research. The findings of this study have implications for non-key universities and their academics on how to effectively promote teaching-research integration.

## 1. Introduction

As early as the 1970s in Britain, the appeal for “teachers as researchers” has been claimed by Lawrence Stenhouse. Many seminal studies in TESOL came into existence due to this initiative (Mckinley, 2019). A holistic academic (Macfarlane, 2011) should achieve a balance between “teaching-informed research and research-informed teaching” (Mckinley, 2019, p. 2).

Chinese college English teachers (CETs) are those who give English lessons to non-English majors in China (Liu and Borg, 2014). Due to the university expansion and the proposal for the massification of higher education initiated by the Chinese government at the end of the last century, there was an urgent need for CETs since English has long been adopted as the major foreign language in China. Many university undergraduates and later master degree holders were enrolled as CETs owing to their language ability rather than academic excellence (Wang and Wang, 2011). With an innate deficiency in academic literacy and an intensive teaching load, they are subjected to a state of struggle and dilemma due to the research-output obsession brought by the contemporary university managerial revolution (Amaral et al., 2003) and regulatory evaluation scheme (Huang and Guo, 2019). To be a teaching-oriented craftsman or a research-oriented teacher is really a problem (Liu, 2011).

As indicated by the iceberg theory of teachers’ practice and cognition (Borg, 2019), university teachers’ practices of research and teaching are influenced by the huge mass of the iceberg underneath, i.e., their beliefs on the two (Hu et al., 2015). The congruence of goal pursuit between research and teaching is impacted by the academics’ perception of their nexus (Daumiller and Dresel, 2020). The contradiction between teaching and research can be better addressed only if a teacher has built an appropriate perception of their relationship (Meng and Chen, 2015).

Although the unity of teaching and research had been proposed by Humboldt in the early 19th century (Ash, 2006) and the teaching-research nexus has long been a focus of research in higher education, language teachers’ conception of their relation has been rarely discussed in applied linguistics (Allison and Carey, 2007; Hu et al., 2015), especially those practitioners in teaching-oriented non-key universities, which undeniably account for a significant portion of the world’s tertiary education. Previous studies on language teachers’ perceptions of teaching-research relations tend to be restricted within the current *status quo* through snapshot-style data, and there is a dearth of research on its development. The longitudinal study is necessary since teachers’ perception of the two varies over time, and it is also very important to identify the impacting factors on such perceptions (Taylor, 2008). This narrative inquiry traces the trajectory of a Chinese CET’s conceptual development in the teaching-research nexus and the impact of such a cognitive process on her professional development from the perspective of sociocultural theory (SCT). It hopes to offer some implications for university administrators who try to promote the two missions among the staff.

## 2. Literature review

### 2.1. The debate on the teaching-research nexus

The debate on teaching-research relations has lasted for more than a century since modern universities came into being in the 19th century (Zhang and Shin, 2015; Tight, 2016). In the early 1990s, Ruth Neumann’s study on senior academic administrators proved the complementary, symbiotic, and mutually enriching nexus between teaching and research. He identified the three simultaneously existing and interrelated levels of the teaching-research nexus, namely, tangible, intangible, and the global level, which transcends individual academics to the departmental plane. Griffiths (2004) and Healey (2005) further delineated the nexus by defining teaching as research-oriented, research-led, research-based, or research-tutored.

However, the following empirical studies have been controversial. Hardly no or weak relation has been proved between research and teaching, the narrow definitions of teaching and research by statistical output measures can be blamed for previous quantitative research, later qualitative studies found the relationship was rather complex (Taylor, 2008; Tight, 2016). Growing research tendencies in the new century have testified to the mutually beneficial relation between research and teaching (e.g., Cadez et al., 2017; Xu and Lei, 2020).

### 2.2. The conflicts between teaching and research

Universities’ competition in research productivity to raise their respective world ranking resulted in the bifurcation of teaching and research (Shin and Kehm, 2012; Burns, 2013; Mckinley, 2019). Too much emphasis has been laid on research activities over quality teaching due to the promotion policy and the sake of institutional funds (Coate et al., 2001; Taylor, 2008; Burke-Smalley et al., 2017; McKinley et al., 2021). The lowest priority has been given to teaching by academics at three university levels (teaching-strong, research-strong, and balanced) in the United Kingdom, which has undervalued the teaching-research synergy (McKinley et al., 2021). One research-focused Slovenian university was also found to prioritize research output over the quality of teaching in a cross-disciplinary study (Cadez et al., 2017). The symbiotic relationship between teaching and research has become an unattainable ideal (Lai et al., 2014).

In language learning, teaching-research conflicts also exist between two isolated groups of stakeholders, the researchers, and the practitioners, who represent research and teaching, respectively, and need mutual understanding and collaboration (Nguyen et al., 2022; Sato and Loewen, 2022). A lack of interface with research has been found in a study among foreign language teachers in the United Kingdom (Marsden and Kasprowicz, 2017). After teaching has become routine, some tertiary EFL teachers seem to be reluctant to get access to research (Sato and Loewen, 2019). L2 teachers’ limited engagement with and in research (Nguyen et al., 2022), full workload, and lack of institutional support (Borg, 2013; Sato and Loewen, 2019) have deepened the gap between teaching and practice.

Although China has also been influenced by the global trend in higher education (e.g., Lai et al., 2014; Huang, 2018), it launched the initiative to support research capability at the end of the 20th century, which was later than its western and northeast Asian counterparts (Shin and Kim, 2017). Research is not the critical basis of university teaching in its teaching-focused system (Shin and Cummings, 2013). Thus, academics in China have a relatively lower perception of the teaching and research nexus, especially in teaching-focused universities except those included in the 985 project (Shin and Kim, 2017). Compared with the strength in teaching, research has long been a conventional weakness for English as a Foreign Language (EFL) practitioners (Dai, 2009; Wang, 2018). In contrast to other disciplines in humanity and social science, the EFL academics’ achievements in scientific research are rather less, especially in the aspects of originality and quality (Wang and Han, 2011). As a major part of Chinese tertiary EFL practitioners, CETs are the most disadvantaged group (Huang and Guo, 2019) in the institutional and national appeal for research output since they lack a solid educational background and sufficient research training (Wang, 2018). Moreover, the switch to mass higher education in China has resulted in tension between teaching and research due to the heavier workload of tertiary level professionals (Zhang and Shin, 2015).

### 2.3. Academics’ perceptions of the teaching-research nexus

The participants in the studies of the perceptions/understandings/conceptualizations of the teaching-research nexus ranged from senior academic administrators (e.g., Neumann, 1992) to university academics (e.g., Brennan et al., 2019) to students (e.g., Buckley, 2011). Most of them believe in the symbiotic and synergistic nexus between teaching and research (Farcas et al., 2017; Brennan et al., 2019; Sippel and Sato, 2022), which seems to contradict the empirical research results on the research-teaching nexus (Hu et al., 2015). The teaching-research nexus is normatively strong but empirically light (Coate et al., 2001; Huang, 2018). According to the participant’s perception, research enhances and updates teaching by integrating frontier knowledge into the teaching materials and engaging students in research (Taylor, 2008; Farcas et al., 2017; Brennan et al., 2019; Sato and Loewen, 2019). Active researchers can instill a questioning and critical way of thinking in the students and share research experiences with them (Neumann, 1992). Although there is a dearth of studies providing concrete evidence of the positive effects of teaching on research (Elken and Wollscheid, 2016), participants in existing studies reported that teaching can widen the academics’ horizons and the contact with the students is stimulating to the academics (Coate et al., 2001; Taylor, 2008; Brennan et al., 2019).

Teaching has long been isolated from research in Chinese tertiary EFL teachers’ conceptions. They seldom read published research (Hu and Tang, 2012) and fail to recognize the facilitative role of research in teaching (Allison and Carey, 2007; Barkhuizen, 2009; Borg and Liu, 2013; Liu and Borg, 2014). Many EFL teachers view themselves as teachers rather than researchers, which has severely impeded their professional development (Wang, 2018). Most Chinese CETs perceive that for their profession, teaching is more important than research (Chen and Wang, 2013) because they seem to have misunderstood research as what produces some obscure theories, which are far beyond the actual teaching practice. Some even hold a narrow view that research engagement just equals academic paper writing or publication and is not helpful for teaching. Such misconceptions just collide with the increasingly prevailing call for research capacity and finally result in cognitive dissonance, especially among those CETs who are going through a transition from the teaching culture to the researching one (Bai, 2018). In fact, research can be divided into theoretical and applied research, both of which can facilitate teaching directly or indirectly (Wen, 2004; Sato and Loewen, 2022). Teaching and research are not two opposing poles since the inquiry of teaching is also a kind of research (Boyer, 1990). On the contrary, few teachers have realized the necessity and importance of the original research based on teaching practice together with teachers’ and students’ development (Meng, 2011). Their research engagements are usually driven by institutional requirements and professional promotion rather than the intrinsic needs for professional development (Hu and Tang, 2012).

### 2.4. The cognitive development of the teaching-research nexus

The deeper reason that has contributed to the research-teaching divide is L2 teachers’ diverse levels of cognition and action toward research (Nguyen et al., 2022). The possible solution to bridge the gap is through teacher education programs (Nassaji, 2012; Nguyen et al., 2022; Sato and Loewen, 2022), which can cultivate teachers’ research mindset and then link teaching and research.

Empirical research on teaching/practice-research connection in the L2 field is a very recent phenomenon (Sato and Loewen, 2022). Among the recent studies, Nguyen et al. (2022) have proved that participating in the inquiry-based instructed second language acquisition course is conducive to cultivating pre- and in-service L2 teacher learners’ cognition of research and its pedagogical effects and finally achieving the synergy between research and practice. Similarly, by involving L2 Spanish teachers in a written corrective feedback writing project, Leow et al. (2022) found that this active collaboration between researchers and teachers has boosted their perception of the researcher-teacher and research-curriculum connection and in turn promoted their personal and professional development as rationale-based practitioners in their lesson plans. The reading group community of practice supported by researchers can help practitioners address their teaching issues with relevant research (Abbott and Lee, 2022). It is also noteworthy that the action research facilitated by teacher educators can foster the collaboration between researchers and teachers and assuage the gap between the two parties (Banegas and Gerlach, 2021; López-Gopar et al., 2021; De Costa et al., 2022).

A better perception of research and practice can not only promote university professionals’ development but also help them to reconstruct their identity. Under the global trend of pushing university practitioners to do research, CETs’ cognitive processes tend to undergo a dramatic change during the reform period (Liu, 2011), which will influence how they position themselves in relation to the reforms and their new identity formation (Golombek and Doran, 2014; Yuan and Lee, 2015; Huang and Guo, 2019), especially the experiences of cognitive contradictions and tensions in the transformation of teachers’ professional identities (Cross, 2010; Xu, 2013; Bao and Feng, 2022). The mutually reinforced relationship between teaching and research can help CETs to alleviate the growing academic stress and construct their professional identities (Huang and Guo, 2019). The CET, Lang, in Bao and Feng (2022), clarified her identity confusion after getting her epiphany on teaching-research congruity. She finally regarded herself as a researcher, besides the long-established teacher identity.

After an overview of the previous studies, much work has been done in investigating the current *status quo* of the teaching-research nexus and the perceptions of the two by related stakeholders. Among the limited research on linking teaching and research, the focus has been put on experimenting the strategies to bridge the teaching-research gap. There is a paucity of research to trace the practitioners’ cognitive development in the teaching-research nexus, especially the thick description of a typical case.

The possible reason may be teachers’ development in cognition is rather intricate and takes time. Research has proved that the study of teachers’ complex inner worlds should be situated in their social context (Johnson, 2009; Gu et al., 2013; Zhang and Zhan, 2014; Kubanyiova and Feryok, 2015). Language teachers’ cognitions are shaped by their personal experiences, such as their educational background, teacher’s educational experience, and the external contexts, such as cultural beliefs and professional context (Taylor, 2008; Golombek and Doran, 2014; Kubanyiova and Feryok, 2015). Since the 1990s, language teacher cognition research has gone through a shift from the individualist cognitive paradigm (e.g., Johnson, 1992; Borg, 1998) to social (e.g., Kubanyiova, 2006; Yuan and Lee, 2014), and then to sociocultural (e.g., Tsui, 2003; Feryok, 2012; Golombek and Doran, 2014). Simon Borg, a leading expert in teacher cognition, also acknowledged that the sociocultural approach, i.e., the deeply situated, complex, and action-oriented view of teacher cognition, had broadened the intellectual horizons of teacher cognition research (2019). Thus, the SCT will be used as the theoretical framework of this study in order to enrich the perspective (Burns et al., 2015) of describing the participant language teacher’s cognitive development in the teaching-research nexus.

## 3. Theoretical framework

**FIGURE 1:**
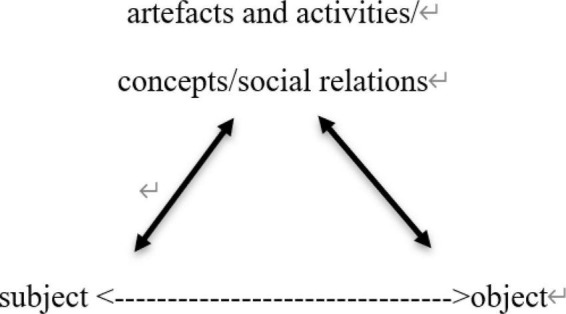
The mediate nature of human/world relationship (adapted from Lantolf and Thorne, 2013).

Sociocultural theory was raised by the Soviet psychologist Vygotsky in the 1930s to explain the development process of human cognition. Mediation is the core concept in SCT (Lantolf and Thorne, 2013). The agents engage in social activities to achieve their goals (i.e., the objectives) *via* mediational tools. All human beings’ higher mental processes in cognition are achieved through sociocultural activities regulated by physical and symbolic means (Swain et al., 2018). Johnson (2009) further classified the mediational tools into cultural artifacts and activities, concepts, and social relations (Figure 1). “Cultural artifacts” are textbooks, courseware, literature, etc. “Activities” are teacher training, reflections, social interaction, etc. Concepts can be divided into everyday (spontaneous) and scientific concepts, both of which have their respective strengths and interact with each other (Vygotsky, 1986). As for teachers, “everyday concepts are their understanding and generalization of their daily teaching experience, and belong to their simple teaching knowledge. Scientific concepts are the systematic generalization and summary of the relevant theory and research outcomes of the discipline, and are the learning materials of teacher education” (Zhang and Yang, 2016, p. 244). “Social relations” mainly refers to the regulation of capable others and the community in the agents’ social interactions.

From a sociocultural perspective, the higher level of human mental functions in the individual will undergo a transformation from the interpsychological plane to the intrapsychological plane and finally come to the stage of internalization, i.e., a transition from other/tool regulation to self-regulation (Vygotsky, 1981). A teacher’s cognition originates from external social interaction. The change in social activities will bring about a change in cognition (Johnson, 2015; Johnson and Golombek, 2020), which is regulated by constant mediation [e.g., the mediation of social relations, concepts, and language learning resources in Ngo (2018); the dialogic interactions between teacher educators and teachers in Johnson (2015); the interscholastic cooperative groups in Meng et al. (2018)] and finally becomes the inner psychological tools for teachers’ thinking. The significance of a mentor’s guidance to struggling academics has been proved by many studies (e.g., Taylor, 2017; Zhang et al., 2017). Under the mediation of the research community, EFL teachers gradually realize the facilitating role of research in teaching and began to understand teaching better (e.g., Meng and Chen, 2019). Their professional development in research and teaching is enhanced through interaction with others (e.g., Lassonde and Israel, 2009; Wen and Ren, 2012; Zhang et al., 2017).

Agency is “the socio-culturally mediated capacity to act purposefully and reflectively on one’s world” (Rogers and Wetzel, 2013, p. 63). Teacher agency plays a critical role in sustaining teachers’ professional development (Tao and Gao, 2017). An agentic teacher will actively participate in research in pursuit of pedagogical improvement and professional development rather than meet the institutional requirement passively (e.g., Meng and Chen, 2019). In EFL education, the teachers’ agency determines the process of teaching-research integration (Xu and Lei, 2020).

Starting from the perspective of SCT, this study explores a Chinese CET, Yuxi^1^’s cognitive process in the teaching-research nexus based on her experience as a visiting scholar at a key university in China and 3 years of teaching and research experiences after visiting study. The article is designed to answer the following four questions:

(1) How did Yuxi perceive research and teaching before her visiting scholar program?
(2) What kinds of changes have Yuxi’s perception of the teaching-research nexus gone through?
(3) What are the mediational tools contributing to these changes?
(4) What are the impacts of such conceptual transformation on Yuxi’s professional development?

## 4. Research methodology

The methodology of both the teaching-research nexus (Neumann, 1992) and teacher cognition (Burns et al., 2015; Borg, 2019) research over the past three decades tend to be pluralistic besides the traditional quantitative approach, with a preference toward qualitative studies. Due to its strengths in a longitudinal study in multiple settings (Barkhuizen et al., 2014), a narrative inquiry will be used to trace the participant’s conceptual development in this study.

### 4.1. Narrative inquiry

Clandinin and Connelly connected Dewey’s experiential philosophy with traditional narrative; raised the concept of ‘three-dimensional inquiry space’, including time, man and society, and place; and then proposed the term ‘narrative inquiry’. Narrative inquiry is both a research phenomenon and a methodology (Clandinin and Connelly, 2000).

Stories and SCT are natural partners (Taniguchi, 2009). Narrative inquiry is often used to access language teacher knowledge and development in sociocultural ontology (Burns et al., 2015). In understanding people’s psychological development, SCT takes contextual factors into consideration (Swain et al., 2018), while the place is an important dimension in the narrative inquiry (Clandinin and Connelly, 2000). SCT lays emphasis on the diachronic development process of human psychological changes, i.e., the genetic method, looking backward and forward in time (Swain et al., 2018), which is in alignment with the past-present-future time structure in the plot of a narrative (Clandinin and Connelly, 2000). Narrative inquiry emphasizes being engaged in the real life to undergo experience (Clandinin and Connelly, 2000), while according to SCT, the development of a human’s higher mental function is the mediated and internalized result of social interaction (Vygotsky, 1978, 1981).

Teachers’ learning itself has the feature of narrative since all the learning is in the continuum of narrative and the individual’s past knowledge background, present experience, and prospects in the future are intertwined together, the individuality and complexity of which cannot be expressed except narratives (Connelly and Xu, 2008). Narratives can best capture the subtleties of human experience in teaching and learning (Webster and Mertova, 2007). Compared with the once-and-for-all interviews, such a dynamic process is much more convincing, educative, and far-reaching in promoting the initiative to integrate teaching and research. Thus, narrative inquiry just fits the purpose of this study, to trace the participant’s conceptual development in the teaching-research nexus among the situated sociocultural contexts.

### 4.2. Research site

Due to the university expansion, 240 newly established regional undergraduate institutions have successively come into being in China, each holding 10–20,000 college students (Liu, 2012). The quality and qualification of the teaching staff in these universities are relatively lower than those in the provincial or national key universities. Previous studies on the integration of teaching and research were mainly conducted in research-oriented universities (e.g., Farcas et al., 2017; Brennan et al., 2019; Xu and Lei, 2020). The CETs in these young non-key universities, who have been marginalized from the academic frontier, need to be studied and cared for. Their professional development in the teaching-research synergy will to some extent impact the quality of overall education in these universities. The study was conducted in one of these universities (University F) in eastern China, which is transforming from the teaching culture into the researching culture. Just as the general educational background stated about the CETs earlier, the college English teaching unit in University F was mainly dominated by master degree holders and university graduates when the study was conducted.

### 4.3. Participant

Yuxi entered the College English Unit at University F after getting her master’s degree. When the excitement of mounting the platform faded away, she realized the pressing need to publish for the upcoming lectureship review. Then she published several low-quality academic articles with no focus successively in a 3-year time. Looking back on these so-called “research achievements,” Yuxi felt rather ashamed since they were not “research” at all. She determined to work for the “authentic” research, but only to find that she was unable to conduct research at all. In the following years, she had been haunted by the anxiety brought on by her weaknesses in research and the upcoming professional evaluation reform, i.e., every teacher is required to finish his/her academic tasks according to their corresponding professional ranks in every 3-year appraising cycle. Having overcome all kinds of difficulties, Yuxi finally managed to study as a visiting teacher at a key university in China (University M, hereafter MU), supervised by professor Li, a leading scholar in teacher education and second language writing.

### 4.4. The authors’ roles and the research ethics

The first author was Yuxi’s classmate during her study at MU, while the second author also made herself an acquaintance with Yuxi during a domestic academic conference. Thus, it is easy to establish rapport with each other, which has made it possible to collect the data. Yuxi voluntarily took part in this study and signed the written consent. The process of data collection has strictly abided by the research ethics, and the final data analysis was sent back to Yuxi for member check and got her consent for use.

### 4.5. Data collection and analysis

The data collected in this study lasts for 4 years (from September 2018 to June 2022), and the data can be classified into two parts (Refer to Table 1). The first part includes Yuxi’s reflective journals (179 entries), and literature reading notes (120 entries) during her 1-year study (the academic year 2018–2019) at MU, which were written under the requirement of her supervisor. The reflective journals were around Yuxi’s reflection or enlightenment over the critical incidents during her study. As for the reading notes, except for the information related to the literature, Yuxi’s critical comments and self-reflection triggered by the literature were also taken down. The second part is the first author’s interaction notes (about 7,500 words) with Yuxi, either face-to-face (during the visiting study) or on social media (after the visiting study), during her visiting study at MU and another 3 years after her visiting study (from September 2018 to June 2022). Such interaction makes it possible to trace Yuxi’s cognitive development of the teaching-research nexus from a sociohistorical perspective. Data in the first part mainly focus on Yuxi’s conceptual development in the research-teaching nexus and the mediational means. Mainly collected in the following 3 years, data in the second part focus on Yuxi’s professional development brought by the progress in research-teaching perception and its continual advances, due to the longitudinal nature of professional development. Since the research converge on Yuxi’s cognitive development in the teaching-research nexus, which largely resulted from her study at MU, the data cited in this article are mainly from the first part. Furthermore, all the literature mentioned in the extracted reading notes, reflective journals, and interaction notes in this study (altogether 1 monograph and 9 academic articles) were also collected and read in detail to cross-check with the three data sources.

**TABLE 1:** Details of the data.

Data source	Details	Information elicited
**Main data**		
Reflective journals	Written by Yuxi during her study at MU	Yuxi’s conceptual development in research-teaching nexus and the influencing factors
Literature-reading notes	Written by Yuxi during her study at MU	Yuxi’s conceptual development in research-teaching nexus and the influencing factors
Interaction notes	Written by the first author during a 4-year time	Yuxi’s professional development brought by the progress in research-teaching perception and its continual advances
**Supplementary data**		
Related literature	Mentioned by Yuxi in the reflective journals, literature-reading and interaction notes	To cross-check with the main data

During the initial communication with Yuxi, the first author realized that her perception of the teaching-research nexus had troubled her for years, and this was later proved to be a typical thread connecting her conceptual development after reading through the data. Nvivo 12 Plus was used to code the data related to the teaching-research nexus. The two authors read and reread the data attentively and coded the data individually. The divergent coding was discussed and recoded together to achieve intercoder reliability. Next, the data were narrated into several condensed stories under several typical themes along the trajectory of Yuxi’s conceptual development in research-teaching nexus. Then, the stories were sent to Yuxi for confirmation and revision. Finally, the stories were inquired within the key concepts of SCT guided by the three dimensions of narrative inquiry, i.e., time (her past experiences, current status, and future projections), man and society (her cognition and emotions, together with the interactions with the social relations), and place (her work context and the visiting university). The stories were reinquired after Yuxi’s member check. All the data were written in Chinese, and the parts used in this study were translated into English.

## 5. Findings

Findings in this part are presented mainly along the route of Yuxi’s conceptual development in the teaching-research nexus during the 4-year period. Her perception before the visiting study is also added based on the interaction notes with Yuxi. Yuxi’s professional development under the impacts of conceptual transformation is demonstrated in the last section, together with the continual perceptive development in the teaching-research nexus. Analysis of the mediational tools leading to her cognitive development is under the theoretical framework of SCT and the backdrop of the three-dimensional space of narrative inquiry.

### 5.1. Is he who is not research-engaged a bad teacher?

Yuxi has always been a devoted teacher who loves teaching and her students. For years, she has followed the principle, ‘to supply the students a drop of water, you must have a bucket of water ahead’. She had been rather satisfied with her commitment to teaching until the time moved to the sixth year of her profession. A prestigious university president’s speech issued by a WeChat official account aroused her reflection.

“…*A teacher who does not engage in research is not a good teacher at all.” I just questioned this remark when I read it. I was not good at research, but I was a good teacher. Could research contribute to teaching? Quite the opposite, research engagement would distract a teacher’s attention from teaching and result in his negligence of the students (Interaction notes, September 26th, 2018)*.

However, the president’s authoritative statement constantly left Yuxi in thought. Two years later, the president of her university made a similar claim during a research mobilization conference. Increasing research pressure also befell her university, for which Yuxi felt rather panicked. For one thing, she did not have the required literacy to conduct research. For another, she still could not find the significance to do that.

Yuxi’s thought on the teaching-research nexus originated from human mediation in social interaction (Johnson, 2009). She restricted her primary perception of teachers’ work to a simple and pure duty, that is, teaching solely. That president’s speech left her wondering about the relationship between teaching and research, and the call for teacher research from her university rippled over her mind once more. The tension and anxiety brought by those external stress forced her to step on the way of research engagement, although she had not got the sense of researching deep in her mind.

All the experiences during a teacher’s schooldays, pre-service teacher-training phase, and in-service years will exert an impact on his cognition (Kubanyiova and Feryok, 2015). In the conversation with the first author, Yuxi said she did not receive systematic, high-quality academic training during her postgraduate education. That was why she failed to develop a scientific perception of the nexus between research and teaching. In the following pre-service teacher training program, she had been stuffed with lectures on education science, psychology, college teaching theory, and teachers’ professional ethics. The pity was that the rationale of educational research and teaching-research integration was not among the training program. Moreover, the College English Unit to which Yuxi is affiliated is dominated by the female staff, whose daily topics are mainly around family, fashion, etc. rather than academics. There was almost no one there to discuss the perception of research with her. In addition, Yuxi had little chance to attend academic conferences due to the limited financial support of her university. Thus, it is not surprising at all that Yuxi had adopted a binary and opposing conception (Borg, 2006) of the teaching-research nexus. Moreover, her cognition has also been constrained by the traditional Chinese culture that a teacher’s duty is to propagate the doctrine, impart professional knowledge, and resolve doubts. But Yuxi’s responsibility, seriousness, and dedication to being a good teacher frequently left her in thought: What is the actual relation between teaching and research? What is research? What kind of research should I do? Yuxi was rather confused.

### 5.2. Research is derived from teaching

Yuxi arrived at MU with the hope of “searching for the true scriptures of doing research.” She also set a 1-year goal for herself: to build a theoretical foundation, learn the corresponding research methods, fix the research interests, and finally raise her academic literacy. However, after attending a series of experts’ lectures and reading abundant academic literature, Yuxi gradually realized that the research topics which she had been so painstakingly struggling with were just among the daily teaching.

*In the workshop of EFL teacher identity, professor Sun mentioned that a teacher could conduct action research based on the doubts or challenges they come across in teaching. It suddenly reminded me of the most challenging work in my class: organizing group discussion*…*I can do action research when I go back (Reflective journal, November 4th, 2018).*

*I meditated for quite a while after reading He and Shi (2012). I have once reflected on the influence of topics on students’ grades, not only in writing but also in reading comprehension. The boys will perform better in the subject of sports, while the girls in the topic of life…*I have had such reflection in my daily teaching, why not take a step further and conduct some research? (Reading notes, November 7th, 2018).**

The workshop as a kind of cultural activity (Johnson, 2009) and professor Sun’s expert guidance are both powerful mediational tools, which informed Yuxi that the aim of research engagement was to tackle the actual problems in teaching. Looking back on her own teaching, there indeed existed many doubts left unsolved. Years ago, Yuxi had already realized the influence of topics on students’ reading performance. With the mediational regulation of the expert guidance, such “everyday concepts” (Vygotsky, 1986) in her daily teaching further made her realize that she did have some reflections in her own teaching and such reflections could all become the starting point of her research.

A teacher who takes the initiative to develop himself will be active in reflection. Reflection, as a kind of activities, which can lead to teacher learning (Dewey, 1933), also plays an essential role in the development of teachers’ cognition (Gu, 2008). The activities such as teaching journal writing will make their implicit rationales explicit, and then they will examine their cognitive system and try to make it perfect and rational (Zhang and Zhan, 2014). During 1-year study as a visiting teacher, Yuxi regularly wrote reflective journals and literature-reading notes following her supervisor’s advice. She gradually recognized that “research is derived from teaching,” and teaching inquiry is also a kind of research (Boyer, 1990) under the interactive regulation of the experts’ guidance, cultural activities, self-reflection, and everyday concepts.

Before her study at MU, research was separated from teaching in Yuxi’s mind, which forced her to struggle with research topics beyond teaching. There were no experts like professor Sun to guide her in her work context. Although she had come up with some valuable reflections in her daily teaching, they made no sense to her and soon dropped away due to her low research consciousness. Such an adverse environment gave rise to Yuxi’s longing for the guidance of a mentor.

### 5.3. Research can repay teaching

Yuxi majored in English education in her postgraduate study and learned some advanced teaching methods. But all these rationales just faded when she mounted the platform for the first time. What constantly occurred to her mind was the teaching scenario of her former intensive-reading class at the university. She just modeled after her teacher of that course, who literally adopted the grammar-translation method. Later, Yuxi tried out several other methods such as communicative teaching, task-based teaching, blended class, etc., but she always dominated the class. While at MU, after reading the literature listed in her supervisor’s course outline, Yuxi found that the phenomenon, such as her imitation of her former teacher, had long been studied by the researchers and termed as ‘apprenticeship of observation’ (Feryok, 2012, p. 97). Long-term apprenticeship of observation will influence novice teachers’ instruction and restrain them from receiving new teaching rationales. The guidance of her supervisor and continuous literature reading made her constantly reflect on the problems she had encountered in her years of teaching.

*After finishing Johnson (2015), I was just wondering how I had been teaching my students? Did I teach them according to my intuition or my academic knowledge or pedagogical expertise?… *then I realize to some extent, I’m not a qualified teacher at all (Reading notes, December 9th, 2018).**

*Our supervisor assembled us to hold another meeting*…*she concluded that the teaching-research unity was the effective way for our professional development (Reflective journal, December 19th, 2018).*

*After reading a series of dynamic assessment papers written by J. Lantolf, I felt my terrible weakness in such assessment. I couldn’t control myself from correcting my students’ mistakes immediately or recasting directly (Reading notes, December 21st, 2018).*

*Ackoff and Greenberg (2008) claims that in the traditional classroom it is the teacher who learns the most. The teacher’s perception and understanding have been consolidated by self-learning and frequent expression in teaching, while the students are unable to learn that much. I finally understood why my students frequently failed to take in what I had been so painstakingly lecturing on them while I already had them at my fingertips (Reading notes, April 1st, 2019).*

Yuxi went to MU with the original intention to improve her academic literacy. Nevertheless, 1 year’s literature reading made her constantly reflect on her problems with teaching principles, classroom activities, classroom feedback, and teacher identity. The scientific concept (Vygotsky, 1986) “apprenticeship of observation” she got from the literature made her realize that the teaching model she had stuck to for 10 years had been studied by the experts, and she just regretted not having met it sooner. By reading Simon Borg’s studies, she realized that research engagement included “engagement with research’ and ‘in research” (Borg, 2010, p. 391). Her literature reading was a kind of engagement with research, which belonged to research engagement. These scientific concepts enabled her to recognize the feedback effect of research on teaching. Her teaching rationales had already been challenged enormously by the sole engagement with research. “What will be the transformation when I finally engage in research?,” Yuxi wondered. Moreover, her supervisor pointed out the way for CETs: the teaching-research unity, which was gradually internalized into Yuxi’s heart by the integrative regulation of the dialogic interaction with the teacher educators (Johnson, 2015; Johnson and Golombek, 2020), literature-reading activities, and scientific concepts.

In retrospect, Yuxi’s work context was lacking in such a research atmosphere. Just like her other colleagues, she tended to read published research before the academic rank appraisal or research project application. It is impossible for teachers to realize their problems in teaching without access to recent literature. Thus, Yuxi’s teaching rationale made no changes in 10 years.

### 5.4. The ultimate goal of doing research is to be a good teacher

Yuxi has finally realized the significance of the teaching-research unity through 1-year visiting study. Her cognition of the research-teaching nexus has risen to a higher level at the end of that year.

*When chatting with professor Li’s Ph.D. student, Lin, I told her I had been gradually awakened to the teaching-research unity. She asked me, “what’s your final aim to study here?” “For academic literacy,” I answered abruptly. She denied my answer and stated that my final aim should be to improve teaching. Having been meditating upon Lin’s words for several days I suddenly woke up one day: Only when a teacher’s academic literacy finally raised can he recognize his problems in teaching, and then become a much more qualified teacher. I felt a relief instantly*…*and finally agreed with that university president, “He who does not engage in research could not be a good teacher” (Reflective journal, May 13th, 2019).*

During Yuxi’s 1-year academic training activities, the community, led by professor Li together with her master and Ph.D. students, and other visiting teachers, served as a significant mediational tool. Yuxi regularly exchanged ideas with these community members. The obscure concepts in her mind gradually become clarified through constant discussion and shared reading of literature. Yuxi’s perception of the teaching-research nexus further rose to a higher level within the zone of proximal development, i.e., from teaching-research integration to researching for a good teacher. She finally realized the necessity and urgency of research engagement. Lin’s enlightenment fully embodied an individual’s cognitive development regulated by the community in social relations.

At Yuxi’s university, there is no such academic community in which everybody can discuss freely and make progress together. Owing to competition in the academic rank appraisal, her colleagues usually conduct research individually. Even if there exists cooperation, it is only restricted to the superficial level.

### 5.5. I am both a teacher and researcher now

In the middle of the second semester during her study at MU, Yuxi struggled to write an academic paper. Challenging as it was, she managed to finish it during the summer vacation that year. During the rather painful journey of writing, she tried to seek guidance and advice from her supervisor and the Ph.D. students and gain insights from the discussion with other visiting teachers. She found her academic career had gradually and painstakingly started. On returning to her own institution, she immediately set out her reform in teaching and was determined to put what she had learned into practice. Now, rather than just transmitting language knowledge and skills, her teaching is increasingly based on scientific rationales and recent research. She puts more attention to switching input into students’ intake; poses challenging questions that will raise the students’ critical thinking and scaffolds her students to raise their cognition levels. Her teaching design based on what she has learned and read was awarded the second prize in a national teaching contest a year after she went back. In 2-year time, she finished another academic paper based on her assessment reform in teaching and successfully got two research projects at the municipal and provincial levels, respectively. She also delivered her research proposal about formative assessment at an academic conference in the third academic year after her visiting study, which was the first attempt in her career. Now, besides being a teacher, she proudly claimed herself as a researcher at the same time.

*Now, instead of simply leading the students to understand the text, I will ask them to assume the writer’s role and navigate them to analyze the author’s writing skills since writing will be mastered through reading*… *If the students fail to answer a challenging question that I have posed to them, I will scaffold their thinking by a sequence of enlightening questions or direct their attention to the clues in the context. Finally, they will get there and gain a sense of achievement*… *As the academic advisor of several English majors, I assign them weekly reading of literary works. They are also required to take down useful expressions and beautiful lines besides submitting the summary. Since you’d better combine implicit learning and explicit learning to enrich the students’ vocabulary (Interaction notes, October 6th, 2021).*

*I read one sentence in the literature recently, “A university is not worthy of the title unless it engages in both teaching and research. Similarly, the academics that the universities employ are not proper academics if they do not research as well as teach (Tight, 2016, p. 2–3).” I could not agree more with it now. I become much more confident in teaching, since not only do I tell my students what it is, I also tell them why it is it. On several occasions, I identified myself as a researcher (although still a struggling one) besides a teacher (Interaction notes, May 4th, 2022).*

Although she aimed at academic literacy during the visiting study, what Yuxi finally reaped was the double harvest in both research and teaching. The summit of her teaching-research perception was the dual identity of a university academic, and in effect, she has also reshaped herself as a qualified teacher and a promising researcher. The profound theoretical foundation she had been accumulating enabled her to be a good teacher. The experience of teaching in turn made her an emerging researcher. In the last story, research for her is not a forced duty anymore, but a symbol of identity. She also began to argue that the university academics who deserve such a title should balance both teaching and research.

Despite being away from the ideal context at MU, she achieved professional development in both teaching and research, since she got a real sense of teaching-research unity. The mediation successfully switched from other regulations to self-regulation.

## 6. Discussion

This part will be discussed around the following three research questions: Yuxi’s gradual conceptual changes in the teaching-research nexus, the mediational means contributing to such transformation, and the following impacts on her professional development.

### 6.1. The conceptual development in the teaching-research nexus

From contradictory to synergistic, Yuxi has achieved her cognitive development through the link of teaching and research. She used to separate teaching from research, which should be mutually reinforcing and closely related. Consequently, her effort in research was just like the water without a source and doomed to make no progress. Meanwhile, the teaching career to which she had devoted her heart and soul also came to a conservative dead end without feedback from research. Yuxi’s prior perception of the teaching-research nexus can be largely attributed to her limited research engagement (Nguyen et al., 2022) and confirms some of the previous research on CETs (e.g., Borg and Liu, 2013; Liu and Borg, 2014; Bai, 2018; Meng et al., 2018, etc.) while providing a sharp contrast to that of the language teachers’ perceptions in the world’s research-oriented universities (e.g., Sippel and Sato, 2022). Such cognition severely restraints teachers’ professional development.

While at MU, Yuxi gradually recognized that “research originates from teaching, and in turn repays teaching, and finally a university academic should research as well as teach.” The transformation of Yuxi’s cognition has undergone a dynamic, enduring, and winding process in the three-dimension space and reached the synergy of teaching and research, which has proved the effect of teacher education projects and research community on teachers’ mindset cultivation (Meng and Chen, 2019; Leow et al., 2022; Nguyen et al., 2022).

### 6.2. The mediational means conducive to the transformation of teaching-research perception

Yuxi’s development in the perception of the teaching-research nexus can be attributed to the combination of a series of enabling factors, i.e., she exerted the agency to interact with the excellent social context at MU under the regulation of all kinds of mediational means.

#### 6.2.1. Specific sociocultural context

The development of an individual’s higher-level thinking can only be achieved when he actively takes part in the interactions in a given sociocultural and historical context (Vygotsky, 1978). Teachers’ perception of the teaching-research nexus is shaped by the external operating context and the internal constitutional working environment (Taylor, 2008). Yuxi’s prolonged misunderstanding of research, outdated teaching rationales, and contradictory teaching-research nexus were undeniably related to the social context she had been exposed to. A teacher’s cognition cannot be raised automatically but achieved within a specific sociocultural context. The top-ranking academic atmosphere at MU provided Yuxi with a “structured mediated space” (Johnson and Golombek, 2020, p. 117) for her cognitive transformation. This once more proved the shaping influence on cognition of external contexts (Golombek and Doran, 2014; Kubanyiova and Feryok, 2015).

#### 6.2.2. The reciprocal interaction of all kinds of mediational means

As an important finding in this study, Yuxi’s cognition toward the research-teaching nexus is gradually shaped by her interaction with the context under the integrative regulation of different mediational tools, such as social relations, cultural artifacts, and activities. All the mediations are reciprocally and complementarily interacted with each other. The symbiotic relationship between teaching and research can only linger on the superficial level as a cold technical term when CETs are preached to do that. True understanding comes only when they indulge in socially regulated activities under all kinds of mediation (Johnson, 2015; Meng et al., 2018; Ngo, 2018). Initially, Yuxi began to reflect on the relationship between research and a good teacher owing to the two presidents’ appeal while holding several queries. She gradually realized that research came from teaching and could in turn repay teaching through literature reading, cultural activities, everyday concepts emerging from daily teaching, and scientific concepts obtained from the literature. Reinforced by her supervisor’s rationale of teaching-research unity, Yuxi’s cognition finally rose to a higher level due to the interaction with the community members, which enrich the evidence of the impacts of the community of practice (Meng and Chen, 2019; Abbott and Lee, 2022). Her doubts about the relation between research and a good teacher had eventually been solved by constant mediation. The culmination of the cognitive process described in the last story, i.e., the dual missions of a university academic, is finally achieved through her self-regulation.

#### 6.2.3. The exertion of agency

Teachers’ agency plays an important role in their cognitive development in research (Meng et al., 2018). A teacher possessed of agency will actively seek the sociocultural context for the exertion of his agency. Facing the changing norms and structures in the higher education institution, Yuxi transcended her original constraining context to improve her academic literacy at MU and seized all the affordance to listen to the experts attentively, learn from competent others actively, read academic literature widely, and write reflective journals and reading notes earnestly. She strived to reflect on her teaching and research while receiving new knowledge and rationales and finally got a real sense of teaching-research unity.

### 6.3. Professional development under the symbiotic teaching-research perception

Teachers’ cognitive development in the teaching-research nexus will boost their personal and professional development to be informed teachers (Leow et al., 2022), and so is the case of Yuxi. Her engagement with/in research has enriched and stimulated her teaching, and her research was also cultivated and bred in her teaching. With the teaching-research synergy in her mind, she now engages herself actively with and in the research. As a result, her teaching activities become theoretically driven and empirically based, which once more proves the pushing force of research on teaching (Farcas et al., 2017; Sato and Loewen, 2019). Meanwhile, as a dedicated teacher, she also finds many issues in her teaching that are worthwhile to research, which has embodied the vitality brought by teaching to research (Taylor, 2008; Brennan et al., 2019).

As another symbol of professional development, Yuxi has transformed her identity from a panic technician into a confident teacher and a developing researcher during the sweeping high education reform. In the first story, we read about a devoted teacher’s efforts to survive in such a hostile context. At that time, a researcher’s identity was not among her awareness, she was just forced to find a way out under the preaching of two presidents’ claims while harboring an inner battle between research and a good teacher. In the fourth story, she finally came up with a solution to the fight between the two: the purpose of scientific research is to shape a qualified teacher, which is not contradictory but aligned with her original aspiration to be a good teacher. Finally, in the fifth story, she has set up the dual identities as both a teacher and researcher and defended them as the due roles of a university professional. It is obvious to see the impacts of the cognitive process of the research-teaching nexus on EFL practitioners’ professional development, as evidenced by the journey from the repellent teaching-research relation to the active construction of the two identities. Yuxi’s identity formation was the natural result of her cognitive development. This resonates with the findings of Huang and Guo (2019) and Bao and Feng (2022).

Every individual’s cognitive development is a continuum. A person’s current status can only be understood when combining his learning and working experiences in the given sociocultural context. His current beliefs and behaviors can in turn serve as the mediation for his future development. Not having received high-quality academic training during the pre-service education results in Yuxi’s superficial, biased perception of research, which has no chance to make a change due to the shortage of academic atmosphere in her in-service context. The final progress in the cognition of the research-teaching nexus achieved at MU will literally lay a foundation for her future teaching and academic development.

## 7. Conclusion

Based on a Chinese tertiary EFL practitioner’s lived experiences when the regulatory evaluation scheme finally came to the teaching-focused non-key universities, this study explores her conceptual development in the teaching-research nexus and its impacts in a 4-year time through a sociocultural lens. Being anxious and panic at the changing external context, she decided to make a change but was trapped by the conflicted perception of teaching and research. Driven by her intrinsic agency and regulated by several complementary mediations, she was finally enlightened: it was in fact not an absolute choice of either teaching or research but a harmonious coexistence of the two, which finally enabled her to achieve professional development successfully. Such a transformation is based on the past and will lead her to the future. Only when CETs clarify the nexus between teaching and research and raise their research consciousness, can they actively engage in teaching research and ultimately achieve mutual development in both teaching and research. This study proves that a mutually reinforced perception of the teaching-research nexus could help CETs relieve the growing pressure to conduct research (Huang and Guo, 2019) and serve as a better solution to respond to the call for teaching-research integration from the institution compared with the external prescriptive requirement.

As a response to the call of Borg (2019) for the implications of individual teachers’ cognition research on the broader enterprises of language teacher education and professional development, the study may shed light on EFL teachers’ professional development in many non-key universities in China or other countries. Admittedly, these EFL teachers should take the initiative and exert their agency to achieve their own professional development. It may also provide insights for administrators and policymakers in some higher education institutions who are painstakingly promoting managerial reform. They should employ proper strategies to enable the staff to realize the symbiotic relation between teaching and research with corresponding policies and financial support. Only in this way can they handle these two missions properly and then be committed to pushing the integration forward. Teachers’ perception of the teaching-research nexus is closely related not only to their own professional development but also to their institutional and sociocultural context. The coordination of these three parties contributes to teachers’ professional development (Gu et al., 2013). Moreover, this study further proves that teaching-research unity is the best way for CETs’ professional development.

This study is not without its limitations. It only focuses on one CET, whose educational background and special working experience may not be representative of all the EFL staff in non-key universities. Nevertheless, the thick description of this teacher’s developmental trajectory can stimulate the initiative of the EFL teachers who are still forced to tackle the required identity in the transition into the researching culture.

## Data availability statement

The raw data supporting the conclusions of this article could be made available by the authors at the readers’ requests. They are not publicly available due to signed consent of the research participant.

## Ethics statement

Ethical review and approval was not required for the study on human participants in accordance with the local legislation and institutional requirements. The patients/participants provided their written informed consent to participate in this study.

## Author contributions

HN conceived the idea, collected the data, analyzed the data, and wrote the manuscript. XW analyzed the data and provided critical feedback. Both authors revised and approved the final manuscript.

## Footnotes

1 Yuxi, professor Li, professor Sun, Lin, University M, and University F in this study are all pseudonyms.
